# Bioinformatics analysis of paravertebral muscles atrophy in adult degenerative scoliosis

**DOI:** 10.1007/s10974-023-09650-8

**Published:** 2023-05-20

**Authors:** Zhigang Rong, Zhong Yang, Chengmin Zhang, Rongxi Pu, Can Chen, Jianzhong Xu, Fei Luo

**Affiliations:** 1 Department of Orthopaedics, Southwest Hospital, Third Military Medical University (Army Medical University), No. 29, Gaotanyan Street, Shapingba District, Chongqing, 400038 People’s Republic of China; 2 Department of Pharmacy and Laboratory Medicine, Third Military Medical University (Army Medical University), Chongqing, 400038 People’s Republic of China

**Keywords:** Adult degenerative scoliosis, Paravertebral muscles, Proteomic

## Abstract

Paravertebral muscles (PVM) act as one of the major dynamic factors to maintain human upright activities and play a remarkable role in maintaining the balance of the trunk. Adult degenerative scoliosis (ADS) has become one of the important causes of disability in the elderly population owing to the changes in spinal biomechanics, atrophy and degeneration of PVM, and imbalance of the spine. Previously, many studies focused on the physical evaluation of PVM degeneration. However, the molecular biological changes are still not completely known. In this study, we established a rat model of scoliosis and performed the proteomic analysis of the PVM of ADS. The results showed that the degree of atrophy, muscle fat deposition, and fibrosis of the PVM of rats positively correlated with the angle of scoliosis. The proteomic results showed that 177 differentially expressed proteins were present in the ADS group, which included 105 upregulated proteins and 72 downregulated proteins compared with the PVM in individuals without spinal deformities. Through the construction of a protein–protein interaction network, 18 core differentially expressed proteins were obtained, which included fibrinogen beta chain, apolipoprotein E, fibrinogen gamma chain, thrombospondin-1, integrin alpha-6, fibronectin-1, platelet factor 4, coagulation factor XIII A chain, ras-related protein Rap-1b, platelet endothelial cell adhesion molecule 1, complement C1q subcomponent subunit A, cathepsin G, myeloperoxidase, von Willebrand factor, integrin beta-1, integrin alpha-1, leukocyte surface antigen CD47, and complement C1q subcomponent subunit B. Further analysis of the Kyoto Encyclopedia of Genes and Genomes pathway (KEGG) and immunofluorescence showed that the neutrophil extracellular traps (NETs) formation signaling pathway plays a major role in the pathogenesis of PVM degeneration in ADS. The results of the present study preliminarily laid the molecular biological foundation of PVM atrophy in ADS, which will provide a new therapeutic target for alleviating PVM atrophy and decreasing the occurrence of scoliosis.

## Introduction

With the aging of the population in the world, the incidence rate of adult degenerative scoliosis (ADS) is increasing every year. ADS is mainly manifested in the form of deformity, low back pain, lower limb radicular pain, and intermittent claudication, which has had a serious negative effect on the quality of life of elderly people (Glassman, et al. 1976). ADS refers to the lateral slip, rotation, and subluxation of the vertebral body caused by the asymmetric degeneration of intervertebral discs and facets in adults, which results in scoliosis with a coronal Cobb angle of > 10°. It is usually found in middle-aged and elderly people over the age of 50, among which the prevalence rate is higher in women than men. This can be associated with postmenopausal osteoporosis and paravertebral muscle (PVM) weakness. ADS can occur throughout the spine, however, it is more likely found in the lumbar vertebra (Buell 2019). During the alterations in lumbar degeneration, the atrophy and fatty degeneration of PVM tissue not only decrease the stability of the lumbar spine and accelerate the lumbar degenerative change but also lead to low back pain (Skorupska 2018).

PVM is divided into a deep layer and a superficial layer. The deep layer is mainly composed of the multifidus muscle, whereas the superficial layer is mainly composed of the erector spinae (Liu et al. 2019). They are an integral part of the spinal dynamic stability system, providing tension belt support for maintaining the balance of the spine and power for the movement of the trunk (Banno et al. 2019). PVM degeneration and atrophy are characterized by a decrease in muscle fiber volume, protein levels, and muscle strength, and an increase in fat infiltration, muscle fatigue, and insulin resistance. These alterations further increase the incidence rate and mortality of several diseases (Cao et al. 2018). Previous studies have shown that the severity of lower back pain (LBP) is closely associated with the degree of multifidus atrophy and fat infiltration (Kader et al. 2000; Hides, et al. 1976; Kjaer et al. 2007). These alterations in multifidus also have different effects on the quality of life of patients with ADS. Recent studies have shown that the increase in trunk muscle fat infiltration and the decrease in muscle volume in patients with ADS are closely associated with the sagittal imbalance of the spine, which indicated that trunk muscle dysfunction is associated with the occurrence and development of ADS (Ferrero et al. 2020). The results of our previous study showed that compared with healthy subjects without degenerative lumbar disease, peak torque values of trunk extensor muscles in the ADS group significantly decreased at three velocity movements (Yang et al. 2020).

However, many of the present studies on PVM degeneration in patients with ADS focused on radiography and physical manifestations, and the molecular biological information of its degeneration and atrophy is not completely known. An in-depth investigation of its biological information alterations will help us understand the potential pathogenesis of degenerative spinal deformity. In this study, we mainly analyze the proteomics of PVM between ADS and non-deformity people (NDP) and investigated the differential expression proteins and related signaling pathways between ADS and NDP. The results of this study will provide a new reference for alleviating paravertebral muscle atrophy, decreasing the occurrence of scoliosis, and improving the quality of life of patients.

## Materials and methods

### Study design and ethical approval

This study was a prospective controlled study and approved by the Ethics Review Committee of the First Affiliated Hospital (Southwest Hospital) of the Third Military Medical University (Ethical batch No. (A)KY202221). Informed consent was obtained from all patients prior to their participation in the study.

### Patients

According to the research needs, we referred to a previous basic research and relevant literature. A total of 6 patients who were diagnosed with ADS and hospitalized in our department were assessed. The inclusion criteria was age > 50 years (including), Cobb angle ≥ 10° on the coronal plane of the full-length X-ray film of the spine, with surgical indications. The relevant preoperative examination results indicated no absolute surgical contraindications, and the patients were deemed ready for posterior scoliosis correction, bone grafting, and internal fixation under general anesthesia. Meanwhile, 6 patients with simple lumbar spondylolysis were designated as the control subjects. The inclusion criteria for this group was age 20–40 years (including) and without scoliosis, lumbar spondylolisthesis, instability, and no obvious degeneration of the intervertebral disc on imaging, and no absolute surgical contraindications. The subjects willingly underwent posterior pedicle spondylolysis bone grafting and screw rod system internal fixation under general anesthesia. The medical records of these patients and their PVM tissue samples during the surgical operation were collected.

### Collection and processing of the PVM samples

After inducing the general anesthesia, the patient were set to the prone position and their lumbar bridge was adjusted. We took the median incision in accordance with the conventional posterior approach, peeled off the PVM on both the sides of the spinous process and along the subperiosteal, fully exposed the screw entry points of the pedicle screws, accurately placed the screws under X-ray monitoring, and then performed spinal canal decompression, bone graft fusion, and orthopedic surgery.

Because the transverse process retractor/automatic retractor was required to be used to pull the paravertebral muscle to both sides during the operation in order to fully expose the operation area, repeated operations during the operation inevitably led to a partial tearing of the PVM. In addition, the operation time was long, which inevitably led to partial PVM ischemia. In order to avoid muscle necrosis affecting the wound healing, such PVM was routinely removed. Therefore, we took the abovementioned PVM tissue samples (approximately the size of soybeans, with a diameter of ≤ 0.5 cm) from each selected case, filled in the sample collection registration form in detail, cleaned the tissue samples with normal saline, placed into a sterile centrifuge tube, and immediately placed them into a liquid nitrogen tank for 10–20 s of quick freezing, wrapped with aluminum foil, and then placed into a -80℃ refrigerator for storage.

### Proteomic detection and analysis

#### Extraction and digestion of protein

Use SDT buffer (4% SDS, 100 mM Tris HCl, 1 mM DTT, pH7.6) to lysis the sample and extract the protein, and use BCA Protein Assay Kit (Bio-Rad, USA) to quantify the protein. Trypsin was used to digest the protein according to the Filter-Aided Sample Preparation (FASP) procedure. Digested peptides from each sample were desalted on C18 Cartridges (Empore™ SPE Cartridges C18 (standard density), bed I.D. 7 mm, volume 3 ml, Sigma), concentrated by vacuum centrifugation, and reconstituted in 40 µl of 0.1% (v/v) formic acid.

#### SDS-PAGE

Mix 20 µg of protein from each sample with 5X loading buffer, and boil with boiling water for 5 min. 12.5% SDS-PAGE gel was used to separate proteins (constant current 14 mA, 90 min), and Coomassie Blue R-250 staining was used to observe the protein bands.

#### Labeling

iTRAQ: According to the manufacturer's instructions (Applied Biosystems), iTRAQ reagent is used to label 100 μg of peptide mixture from each sample.

TMT: According to the manufacturer's instructions (Thermo Scientific), the TMT reagent was used to label 100 μg of peptide mixture from each sample.

#### Strong cation exchange (SCX) fractionation

Use AKTA Purifier system (GE Healthcare) to separate the labeled peptides by SCX chromatography. Before the dried peptide mixture was applied to a PolySULFOETHYL 4.6 × 100 mm column (5 µm, 200 Å, PolyLC Inc, Maryland, U.S.A.), it was reconstituted and acidified with buffer A (10 mM KH2PO4 in 25% of ACN, pH 3.0). Peptides were eluted at a flow rate of 1 ml/min. The gradient was 0% buffer B (500 mM KCl, 10 mM KH2PO4 in 25% of ACN, pH 3.0) for 25 min, 0–10% buffer B for 25–32 min, 10–20% buffer B for 32–42 min, 20–45% buffer B for 42–47 min, 45–100% buffer B for 47–52 min, and 100% buffer B for 52–60 min. After 60 min, buffer B was reset to 0%. The elution was monitored by the absorbance at 214 nm, and the fractions were collected every 1 min. The collected fractions were desalted on C18 Cartridges and concentrated by vacuum centrifugation.

#### LC–MS/MS analysis

Q Exactive mass spectrometer (Thermo Scientific) is coupled with Easy nLC (Thermo Fisher Scientific) for LC–MS/MS analysis, and the analysis time is 60/90 min. The peptides were loaded onto the reverse phase capture column connected to the C18 reverse phase analytical column in buffer A (0.1% Formic acid) and separated using the linear gradient of buffer B (84% acetonitrile and 0.1% Formic acid), the flow rate is 300 nl/min and is controlled by IntelliFlow technology. The mass spectrometer operates in a positive ion mode.

#### Identification and quantitation of proteins

The MASCOT engine (Matrix Science, London, UK; version 2.2) embedded with Proteome Discoverer 1.4 software, which can be used for identification and quantitative analysis, is used to search the MS raw data of each sample.

### Bioinformatic analysis

#### Cluster analysis

First, the quantitative information of proteome is normalized (normalized to (− 1,1) interval). Then use the Complexheatmap R package (version 3.4) to classify the two dimensions of samples and protein expression, and generate hierarchical clustering Heatmap.

#### GO function annotation

The target protein sets were annotated with Blast2GO, which can be roughly summarized into four steps: sequence alignment (Blast), GO entry extraction (Mapping), GO annotation (Annotation) and InterProScan annotation (Annotation Augmentation).

#### KEGG pathway annotation

The KEGG pathway of the target protein collection is annotated with KAAS (KEGG Automatic Annotation Server) software.

#### Enrichment analysis

The distribution of each GO classification (or KEGG pathway or Domain) in the target proteome and the whole proteome was compared using Fisher's exact test, and the target proteome was enriched with GO annotation or (or KEEG pathway or Domain) annotation. Only functional categories and pathways with p-values below 0.05 were considered significant.

#### Protein–protein interaction (PPI) analysis

Based on the information in the IntAct (http://www.ebi.ac.uk/intact/main.xhtml) or STRING databases (http://string-db.org/), the direct and indirect interaction relationship between target proteins is clarified, and the interactive network is generated and analyzed using the CytoScape software (version No. 3.2.1). In addition, the importance of each protein in the PPI network is evaluated by calculating the degree of each protein.

### Establishment of the rat scoliosis model

Because the volume of the PVM tissue samples collected was small, only proteomic analysis was performed. To determine the morphological changes of the PVM in scoliosis compared with those without scoliosis, we constructed a rat scoliosis model based on the research of Lifeng Liu et al. (1976). Nine 4-week old female Wistar rats weighing approximately 60 g were randomly assigned to 3 groups: Group 1 (the control group or the no scoliosis group that did not receive any treatment). Group 2 (the scoliosis group, which received anesthesia with isoflurane inhalation and involved tying with non-absorbable silk thread subcutaneously). In this group, the left subscapular angle and the ipsilateral iliac bone were tied to approximately 80% of the original subscapular angle to the ipsilateral iliac bone, which made the spine slightly right convex, after which the forelimbs were cut off to establish a bipedal rat model. Group 3 (also a scoliosis group, albeit the degree of tethering was approximately 60% from the original subscapular angle to the ipsilateral iliac bone, while the other treatment was the same as that of Group 2). All rats were raised independently. Group 1 used a standard cage, and Groups 2 and 3 used special high cages. After 8 weeks of tethering, the silk thread was cut and the rats were observed for 2 weeks. Next, the rats were executed, and the angle of scoliosis was evaluated by Micro-computed tomography (CT). The PVM was stained with HE staining, Oil red O staining, and Sirius red staining in accordance with the instructions of the Kit, and the morphology of the PVM was analyzed.

### Immunofluorescence

The tissue sections were dewaxed, and then the citrate buffer antigen was repaired by microwave (92–96 ℃, 10–15 min), and then cooled to room temperature naturally. Clean with pure water, dry in circles, and wash with PBS for 3 times, 5 min each time. Use normal sheep serum to seal at 37 ℃ for 60 min, pour out the excess serum, drop the first antibody, and incubate at 4 ℃ overnight. Then rinse with PBS three times (5 min each time), add fluorescein labeled secondary antibody, incubate in dark at 37 ℃ for 60 min, and rinse with 0.01 M three times (5 min each time). The anti-quenching sealing agent is used to seal the tablet, and it is stored in a refrigerator at 4 ℃ away from light. Finally, the fluorescence microscope is used to observe and take photos.

### Western blotting analysis

Radioimmunoprecipitation assay lysis buffer was used to extract the total protein of each group, and then the protein concentration was quantified by the bicinchoninic acid assay. 10% sodium dodecyl sulfate polyacrylamide gel electrophoresis was used to separate the Equivalent amounts of protein (20 mg) in each group, and then transferred onto 0.2 mm polyvinylidene fluoride membranes. The membranes were blocked in 3% BSA (containing 0.1% Tween 20) for 12 h and then blots were incubated (4 ℃ overnight) with primary antibodies against Myeloperoxidase (MPO) (1:1000, ABclonal Technology, China.) and GAPDH (1:2000, SanJian, China). After the membranes were washed with TBST, the secondary antibody (anti-rabbit IgG or anti-mouse IgG diluted at 1:5000) was used to incubated them at room temperature for 1 h. Use the Quantity One program and the Enhanced Chemiluminescence Kit (Amersham Biosciences, UK) to visually analyze the signals on the membranes.

### Statistical analyses

The data were expressed in the form of mean ± standard deviation. Statistical analysis was conducted with two-way analysis of variance using the GraphPad Prism 7.0 statistical software package (GraphPad Software). p < 0.05 was considered to indicate statistical significance.

## Results

### Morphological analysis showed atrophy of PVM in rats with scoliosis

**Fig. 1:**
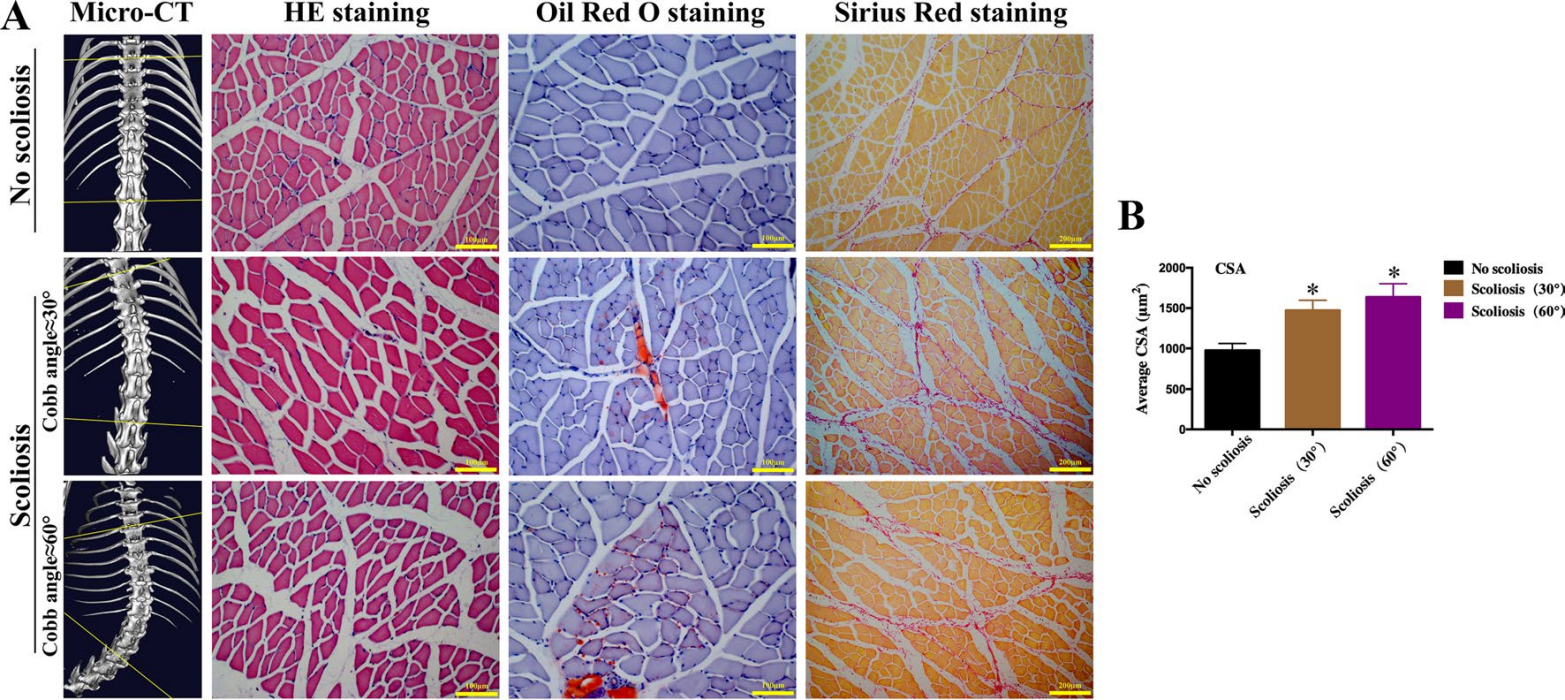
**A** The morphological changes of the PVM in scoliosis compared with those without scoliosis. The volume of muscle fibers, the space between muscle fibers, fat deposition and fibrosis of three groups without scoliosis, with a scoliosis angle of about 30° and a scoliosis angle of about 60° were compared from 3D reconstruction of spinal CT, H&E staining, Oil red O staining, and Sirius red staining of the paravertebral muscles. **B** Comparison of cross-sectional area (CSA) of muscle fibers

We simulated the scoliosis model in rats and then performed 3D reconstruction of spinal CT, H&E staining, Oil red O staining, and Sirius red staining of the paravertebral muscles. The results showed that the PVM of the rats without spinal scoliosis did not undergo atrophy. The volume of muscle fibers did not decrease, the space between muscle fibers did not increase, and there was no distinct fat deposition and fibrosis. However, with the aggravation of scoliosis, the atrophy of PVM became more distinct, the muscle fiber space gradually increased, and the fat deposition and fibrosis became more distinct (Fig. 1A). Meanwhile, compared to the group without scoliosis, the cross-sectional areas (CSA) of muscle fibers in both scoliosis groups increased (p < 0.05, Fig. 1B).

### Data processing and differential protein analysis

**Fig. 2:**
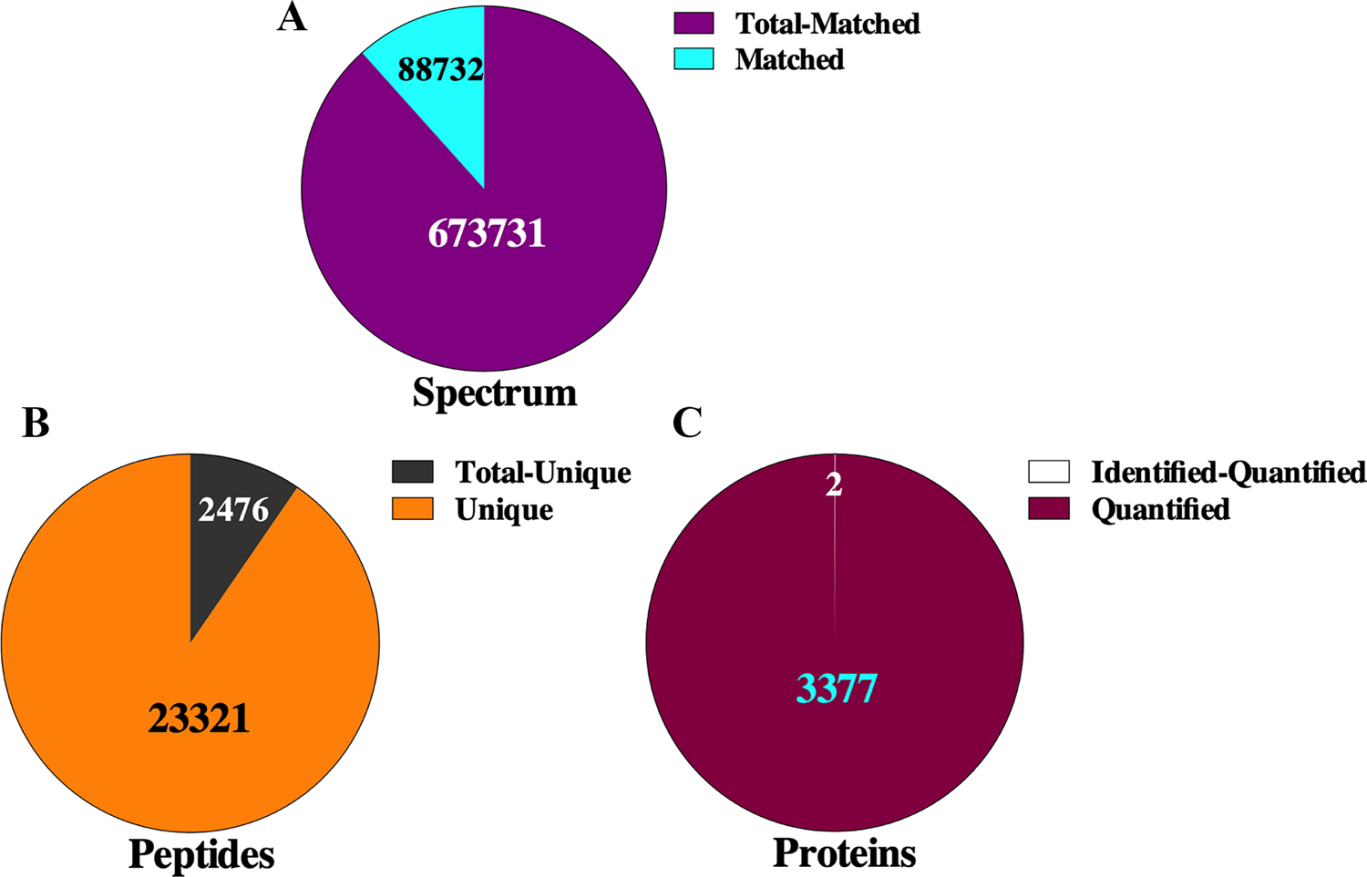
Graphical summary of the number of spectra obtained, the number of peptide segments identified and the number of proteins identified in this study

The number of the total spectra obtained in this study was 7,62,463 (which included 88,732 matched spectra), the number of identified peptides was 25,797 (which included 23,321 unique peptides), and the number of identified proteins was 3379 (which included 3377 quantified) (Fig. 2).

### Differential analysis of protein expression

**Fig. 3:**
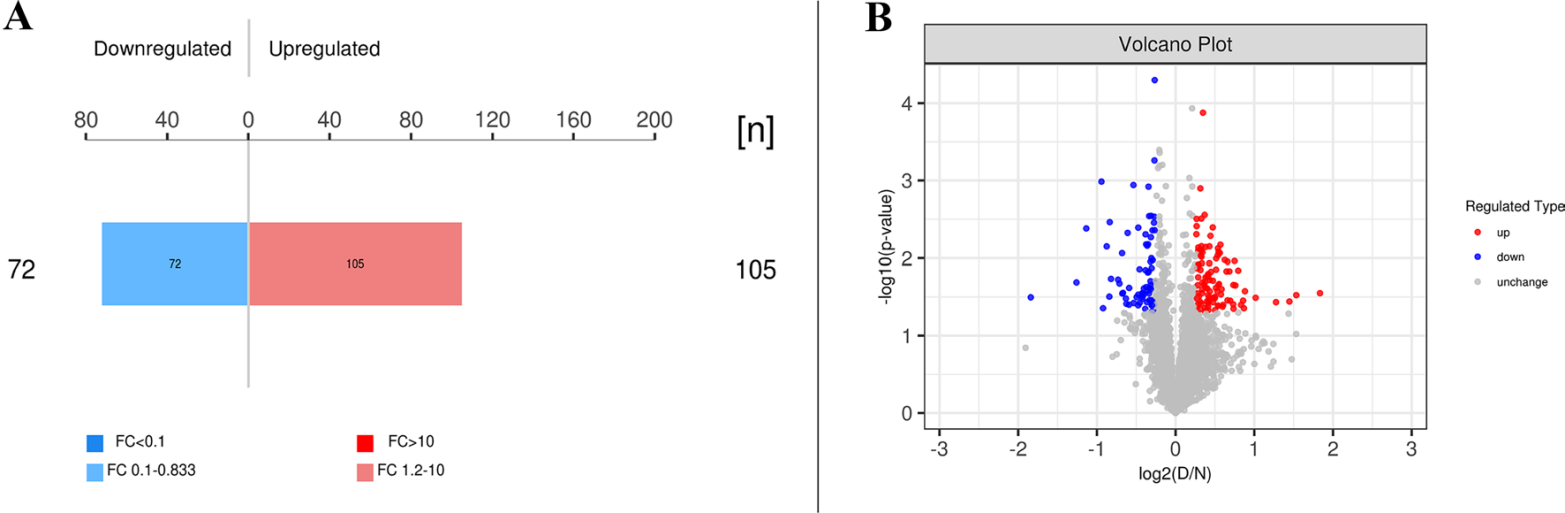
**A** Histogram of protein quantitative difference results. **B** Volcano map of scoliosis group compared with non-scoliosis group. The abscissa was the fold change (logarithmic transformation with 2 as the base), the ordinate was the significance P-value (logarithmic transformation with 10 as the base) of the difference, the red dots in the figure were the up-regulated significant differential expression proteins, the blue dots were the down-regulated specific differential expression proteins, and the gray dots were the proteins with no difference change

To investigate the proteins with different expression in the PVM of patients with ADS compared with patients with simple lumbar spondylolysis, we further screened the experimental data for differences. The number of upregulated and downregulated proteins in the ADS group was determined by taking the expression fold change (FC) > 1.2 times (upregulation > 1.2 times or downregulation < 0.83 times) and P value < 0.05 (*t*-test) as the criteria to screen out significantly different expression proteins as shown in the column of significantly changing in abundance (Table 1). During the same time, the results are presented in the form of a bar chart (the number of proteins with upregulation and downregulation > 10 times is indicated with a darker color) (Fig. 3A). Moreover, to show the significant difference between the two groups of proteins, the proteins in the ADS group were plotted as a volcano map based on the two factors of expression difference fold change (FC) and P value (*t*-test). The significantly downregulated proteins were marked with blue (FC < 0.83 and P < 0.05), the proteins that were significantly upregulated were marked with red (FC > 1.2 and P < 0.05), and the gray were the proteins without a difference (Fig. 3B).

**Table 1:**
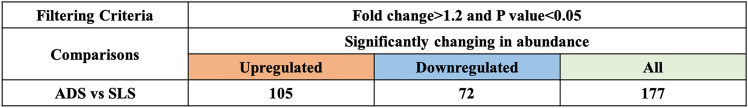
Statistical table of protein quantitative difference results

**Fig. 4:**
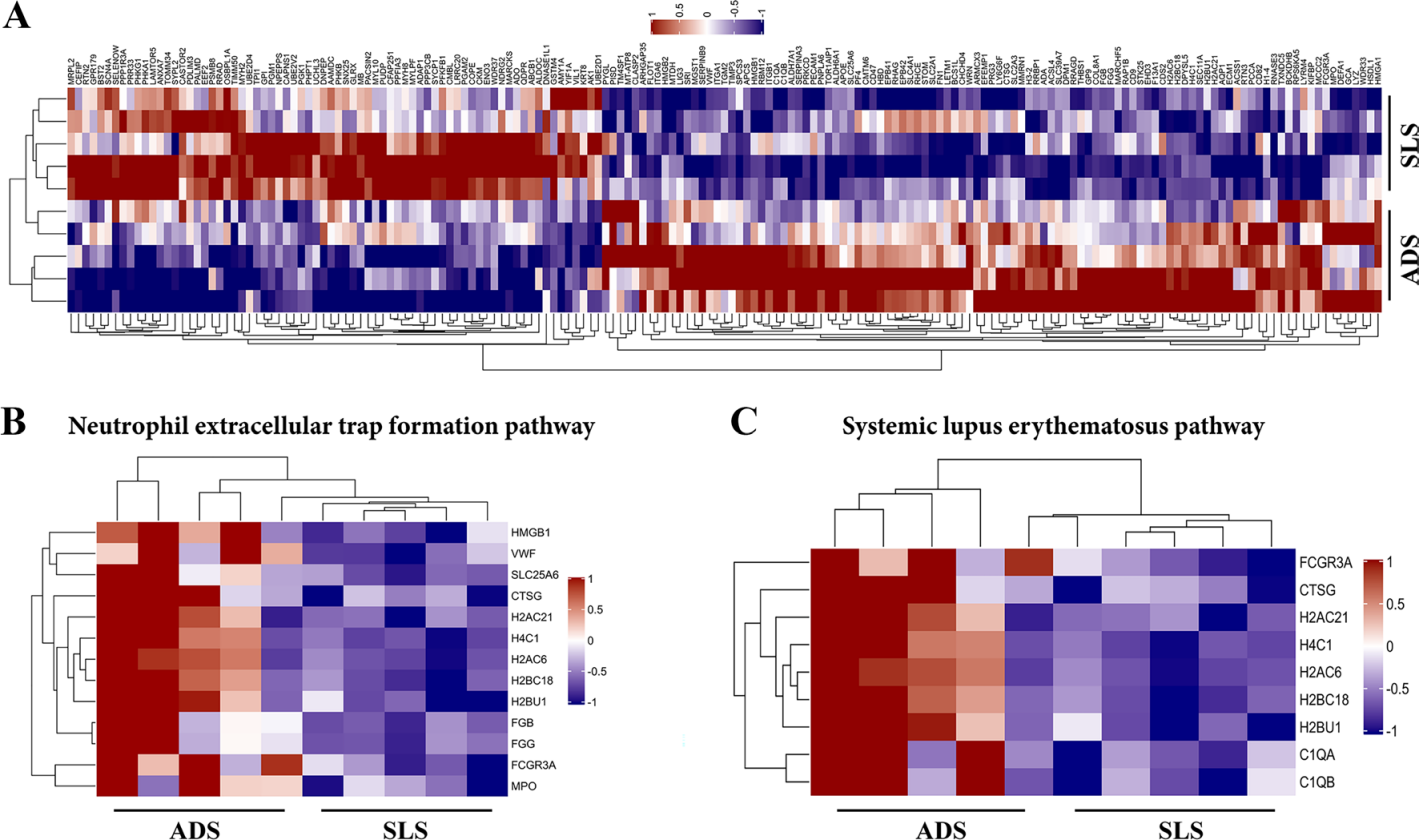
**A** The heat map showed the classification analysis results of the two groups of differentially expressed proteins using hierarchical clustering algorithm. The expression amount of proteins with significant differences in different samples was standardized by log2 method and displayed in the heat map in different colors. Proteins that are significantly upregulated are shown in red, proteins that are significantly down-regulated are shown in blue, and proteins without quantitative information are shown in gray. **B** The heat map of Neutrophil extracellular trap (NET) formation pathway. **C** The heat map of systemic lupus erythematosus pathway

Moreover, to test the rationality of this study grouping, and determine whether the changes of differentially expressed proteins can represent the significant effect of degenerative scoliosis on PVM, we determined the expression patterns of samples between and within the two groups of ADS and patients with simple lumbar spondylolysis, and hierarchical clustering algorithm was used to classify the differentially expressed proteins of the two groups, which were shown in the form of heat maps. The results showed that the differentially expressed proteins obtained by the screening criteria of fold change > 1.2 times and p value < 0.05 (*t*-test) could effectively separate the comparison groups, which indicated that the screening of differentially expressed proteins can reflect the biomechanical changes caused by degenerative scoliosis and the significant effect on the paravertebral muscles (Fig. 4A).

### Go function analysis

**Fig. 5:**
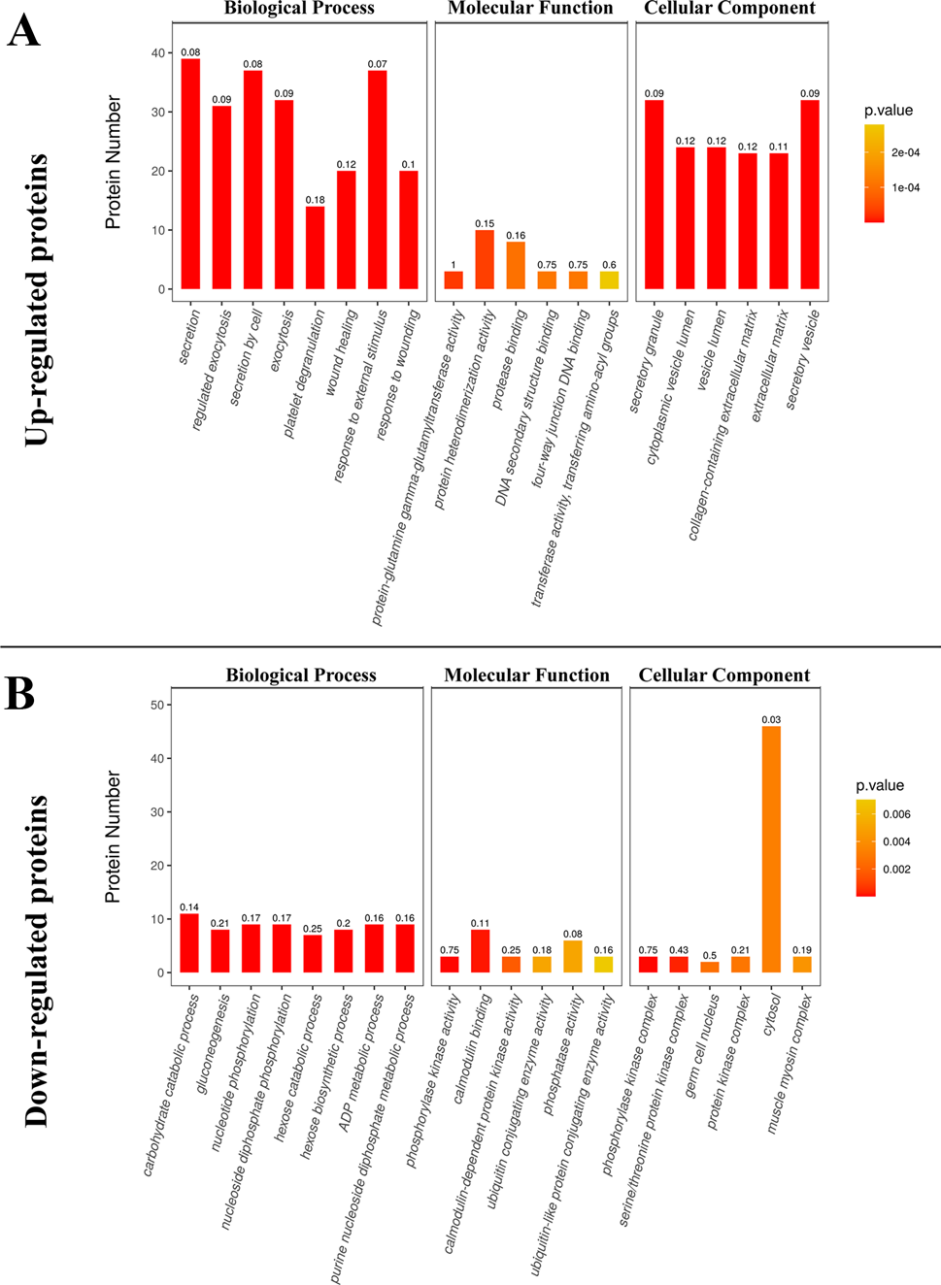
Histograms showed the enrichment of GO items under the three GO categories of up-regulated protein and down-regulated protein. The ordinate in the figure is the protein number (Rich Factor ≤ 1), and the abscissa represents the statistical results of different proteins under each GO functional classification. The numbers on the graph represents the significance of the enriched GO function classification, that is, the P value is calculated based on the Fisher's Exact Test. The number magnitude represents the size of the P value (- log10)

The GO function of all differentially expressed proteins in the present study was annotated using Blast2Go (Gotz et al. 2008). All differentially expressed proteins were compared with all proteins of the reference species (or all proteins identified in the experiment) per the annotation results of the GO function, and the significant differences between the two were analyzed by Fisher’s exact test to find out functional categories enriched in all differentially expressed proteins (p < 0.05). The enrichment of GO items under three GO categories is displayed in a histogram. The results showed that upregulated proteins in the ADS group were closely related to important biological processes (BP) such as secretion, regulated exocytosis, secretion by cells, exocytosis, and platelet degranulation (Fig. 5A). These proteins were also related to molecular functions (MF) such as protein heterodimerization activity, protein–glutamine gamma-glutamyl transferase activity, protease binding, four-way junction DNA binding, and DNA secondary structure binding (Fig. 5B). These upregulated proteins were closely related to cellular components (CC) such as secretory granules, vesicle lumen, cytoplasmic vesicle lumen, collagen-containing extracellular matrix (ECM), and secretory vesicles (Fig. 5C). However, downregulated proteins in the same group were mainly related to BPs such as carbohydrate catabolism, hexose catabolism, gluconeogenesis, hexose biosynthesis, and nucleoside diphosphate phosphorylation (Fig. 5D). They were related to MFs such as phosphorylase kinase activity, calmodulin binding, and calmodulin-dependent protein kinase activity (Fig. 5E) and were associated with CCs such as phosphorylase kinase and serine/threonine protein kinase complexes (Fig. 5F).

### KEGG pathway analysis

**Fig. 6:**
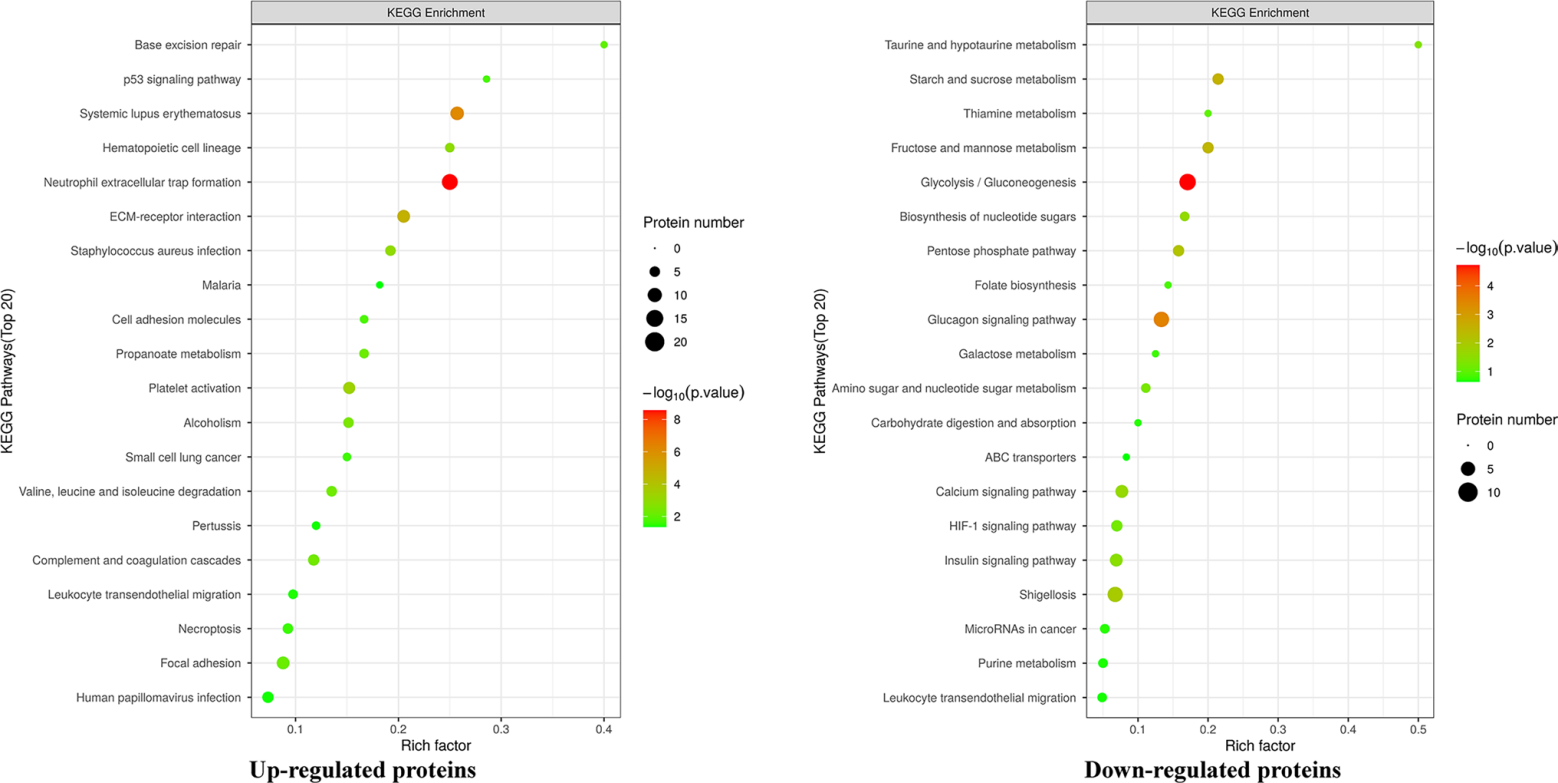
Bubble charts of KEGG pathway enrichment of up-regulated protein and down-regulated protein. The abscissa in the figure is the enrichment factor (Rich Factor ≤ 1), and the ordinate represents the statistical results of differential proteins under each KEGG pathway. The bubble color represents the significance of the enriched KEGG pathway, that is, the P value is calculated based on the Fisher's Exact Test. The color gradient represents the size of the P value (− log10). The closer the color is to red, the smaller the P value, and the higher the significance level of the enrichment of the corresponding metabolic pathway

We compared all differentially expressed proteins with all proteins of the reference species (or all proteins identified in the experiment) per the KEGG annotation results to reveal the overall metabolic pathway-enrichment characteristics of all differentially expressed proteins and find the most significantly enriched KEGG metabolic pathway by evaluating the significance level of the protein enrichment of a specific KEGG metabolic pathway. The results showed that upregulated proteins in the ADS group mainly participated in signaling pathways such as neutrophil extracellular trap (NET) formation, systemic lupus erythematosus, and ECM–receptor interactions (Fig. 6A). In contrast, downregulated proteins were mainly involved in pathways including glycolysis/gluconeogenesis and glucagon signaling (Fig. 6B). Simultaneously, the analysis of the hierarchical cluster algorithm showed significant differences between the ADS and control groups regarding proteins related to the NET formation pathway and systemic lupus erythematosus, especially the former (Fig. 4B, 4C).

### Construction and analysis of the interaction network of differentially expressed proteins

**Fig. 7:**
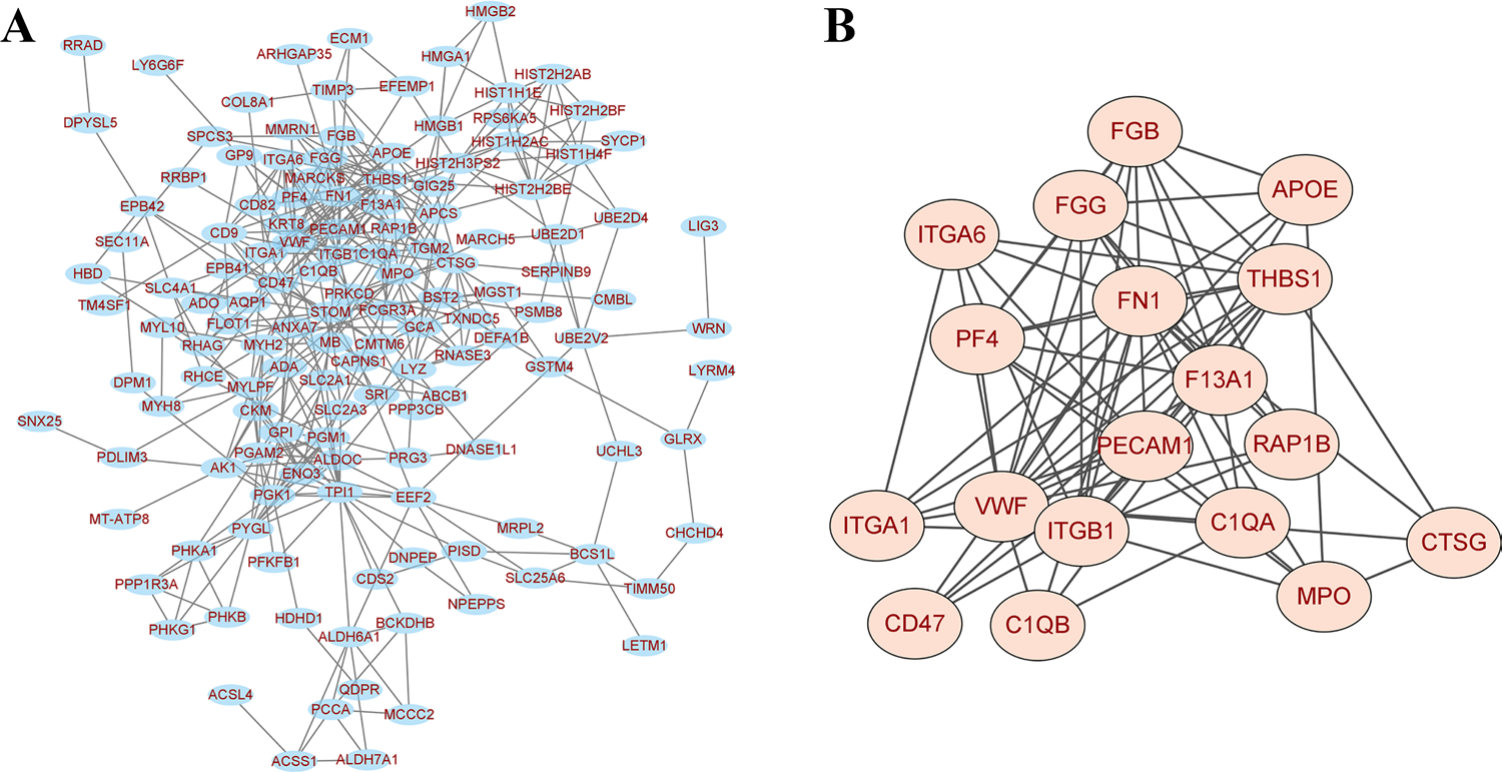
**A** Network diagram of protein–protein interaction (PPI) function classification. **B** Network diagram of the 18 most significant interaction module

Based on the PPI in STRING or IntAct, Cytoscape was used to construct a PPI network for differentially expressed proteins in the ADS and simple lumbar spondylolysis groups in this study. Furthermore, we imported the PPI network constructed by STRING into Cytoscape 3.9.1 for visual analysis. The network consisted of 149 nodes and 435 edges (Fig. 7A). MCODE plug-in was used to analyze the most significant interaction module, which consisted of 18 nodes and 73 edges (Fig. 7B). The aforementioned 18 nodes/proteins were fibrinogen beta chain (FGB), apolipoprotein E (APOE), fibrinogen gamma chain (FGG), thrombospondin-1 (THBS1), integrin alpha-6 (ITGA6), fibronectin-1 (FN1), platelet factor 4 (PF4), coagulation factor XIII A chain (F13A1), ras-related protein Rap-1b (RAP1B), platelet endothelial cell adhesion molecule 1 (PECAM1), complement C1q subcomponent subunit A (C1QA), cathepsin G (CTSG), myeloperoxidase (MPO), von Willebrand factor (VWF), integrin beta-1 (ITGB1), integrin alpha-1 (ITGA1), leukocyte surface antigen CD47 (CD47), and complement C1q subcomponent subunit B (C1QB), and all these proteins were upregulated. Among them, five proteins, VWF, CTSG, FGB, FGG, and MPO, participated in the NET formation signaling pathway. The above-mentioned key proteins and the NET formation signaling pathway indeed played an important role in the occurrence, development, and prognosis of PVM degeneration in patients with ADS.

### Immunofluorescence and Western Blot

**Fig. 8:**
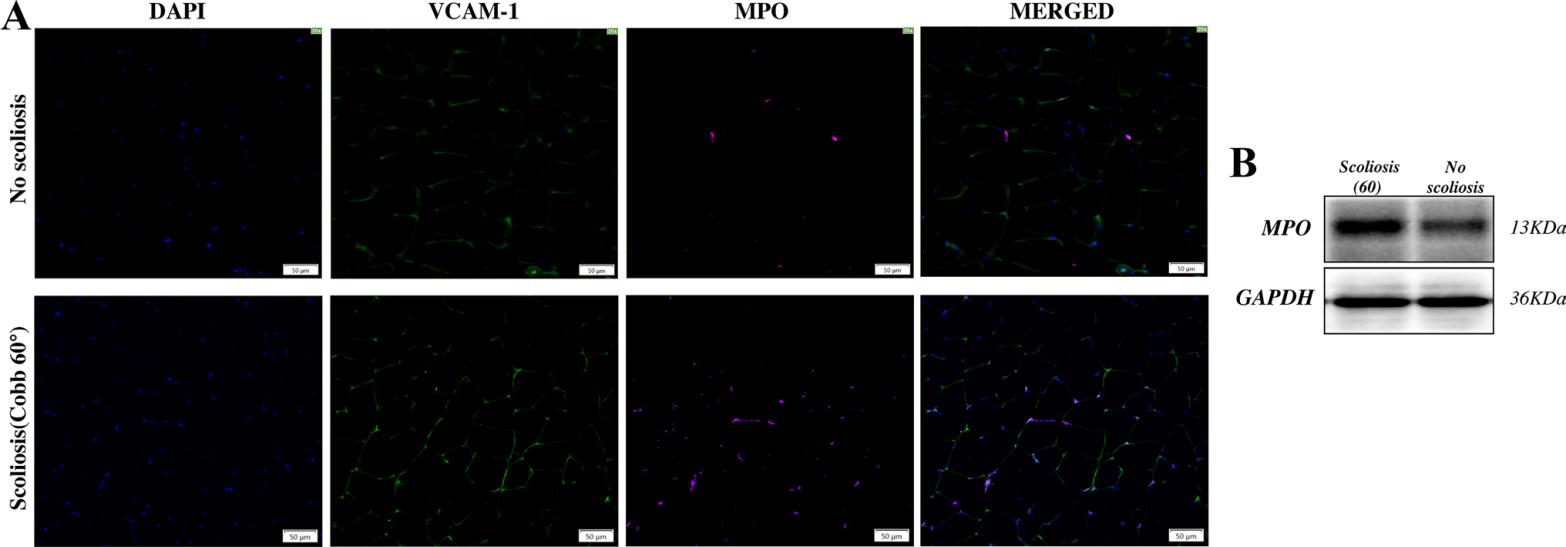
Immunofluorescence and western blot were used to detect the expression of MPO, one of the components of NETs in No-scoliosis group and scoliosis group (Cobb angle about 60°)

To further verify that compared with the no-scoliosis group of rats, the NET formation signaling pathway showed an up-regulated trend in the process of paravertebral muscle degeneration in the scoliosis group (Cobb angle about 60°). We further confirmed by immunofluorescence and western blot that the expression of MPO, one of the important components of NETs, was significantly increased (Fig. 8A, B).

## Discussion

ADS is caused by the asymmetric degeneration of intervertebral discs and facet joints, often accompanied by lateral sliding, the rotation and subluxation of the vertebral body, and the atrophy of PVM. PVM degeneration further aggravates the deformity of the spine. This vicious circle seriously affects the quality of life of the elderly population. So far, many methods have been used to establish animal models of scoliosis for the study of scoliosis, such as tethering or external fixation techniques, the application of rat tail suspension models, unilateral resection of intercostal or dorsal nerve roots, changes in the normal anatomical structure of the spine or ribs, and so on. In this study, the rat scoliosis model is based on the spinal deformity model published by Lifeng Liu et al. in Spine (1976). Meanwhile, we further explored molecular and biological changes during PVM degeneration in ADS using bioinformatics analysis methods to understand the potential pathogenesis of ADS thoroughly. A total of 177 differentially expressed proteins related to PVM in ADS were obtained by proteomics, 105 of which were upregulated, and 72 were downregulated. By constructing a PPI network, 18 core differentially expressed proteins were obtained. Further, the KEGG pathway analysis showed that the NET formation signaling pathway probably played a key role during PVM degeneration in ADS.

Skeletal muscle is a special structure that maintains its homeostasis by balancing functional, physical, and chemical interactions between muscle and myofiber microenvironments, and it can change muscle functions and structures per the amount of activity and mechanical load (Meer et al. 2011). Muscle atrophy is usually easily caused by several factors, including load changes such as microgravity exposure, fracture plaster fixation, rat tail suspension, and long-term bed rest (Lecker et al. 2004; Sandri 2008). In this study, PVM atrophy in ADS was caused by a change in mechanical load after spinal deformity. The morphological test results of the rat scoliosis model showed that more severe spinal deformity was associated with more obvious PVM atrophy and more significant fat deposition and fibrosis. However, the specific mechanism underlying this process is still unclear. Regardless of the initial cause, skeletal muscle atrophy will lead to a typical cascade event regulated by immune system cells (Brunelli and Rovere-Querini 2008). However, the role of immune-related cells in aggravating muscle damage and affecting muscle repair, a crucial aspect of muscle atrophy, has not been studied well (Tidball 2005). After muscle injury, the innate immune system is induced to recruit cells, such as polymorphonuclear leukocytes and monocytes/macrophages, to remove cell fragments and release harmful molecules (Muller 2003). Simultaneously, infiltrating immune cells can continue to survive and be activated, causing tissue inflammation, thus leading to fat accumulation and collagen deposition with fibrosis in skeletal muscles.

Neutrophils are the first line of defense of the immune system against fungal and bacterial infections under physiological conditions. The diversity of their individual activities and participation in BPs (such as NETs) play a critical role in inflammatory responses, overall immune coordination, and disease development (You et al. 2022). Many studies have shown that neutrophils and their migration and infiltration may lead to major signaling events in skeletal muscle atrophy; however, neutrophil invasion into muscles is still controversial to some extent (Dumont et al. 2008; Pillon et al. 2013). In particular, the exact role of neutrophils in common inflammatory conditions in skeletal muscles, such as sterility, non-hypoxia, and non-chronic inflammation, is still unclear. The KEGG pathway analysis in the present study showed that the upregulated proteins associated with PVM in ADS mainly participated in the NET formation signaling pathway. Therefore, we propose a possible hypothesis that after ADS occurrence, PVM tension gradually changes, and muscle cells undergo aseptic damage. Simultaneously, neutrophils intervene in the form of NETs, further affecting muscle homeostasis and BPs, leading to fat deposition or fibrosis, thus aggravating PVM atrophy.

Recently, some in vivo and in vitro morphological studies have shown that neutrophils can aggravate muscle injury and atrophy by releasing free radicals and proteolytic enzymes in the aseptic injury of skeletal muscles (Carden et al. 1990). Studies have shown that myotubular injuries and tension may induce the release of factors that can affect neutrophil chemotaxis and activation; only damaged myotubes will release such factors to activate neutrophils to produce free radicals (Moraes et al. 2006). This indicates that muscle cells will release inflammatory molecules, which can activate neutrophils to varying degrees, and these molecules will change with the type and intensity of injuries (Elbim et al. 1994). Therefore, further understanding the relationship between skeletal muscle atrophy and neutrophils is essential to explore a potential therapeutic target for PVM atrophy.

To conclude, we preliminarily described molecular and biological changes during PVM atrophy and degeneration in ADS, showing signaling pathways that may play a critical role in it. Our findings may reveal a new way to further explain molecular signaling during PVM degeneration, helping to provide new research targets and strategies for treating ADS and improving the quality of life.
